# Modulation of cardiometabolic risk and CardioRenal syndrome by oral vitamin D_3_ supplementation in Black and White Southern Sahara residents with chronic kidney disease Stage 3: focus on racial and ethnic disparities

**DOI:** 10.1080/0886022X.2022.2106244

**Published:** 2022-08-05

**Authors:** Asma Bouazza, Amina Tahar, Samir AitAbderrhmane, Messaoud Saidani, Elhadj-Ahmed Koceir

**Affiliations:** a Nutrition and Dietetics in Human Pathologies Post Graduate School, Bioenergetics, Intermediary Metabolism team, Biology and Organisms Physiology laboratory, USTHB, Algiers, Algeria; b Diabetology unit, Seghir Nekkache Hospital, Algiers, Algeria; c Clinical Nephrology Exploration Unit, Dialysis and Kidney Transplantation Unit, University Hospital Center of Beni Messous, Algiers, Algeria

**Keywords:** Chronic kidney disease, racial and ethnicity, vitamin D, SHPT, cardiometabolic, cardiorenal, RAAS, NT-proBNP

## Abstract

**Objectives:**

Several studies have shown that cholecalciferol supplementation (25OHD-S) in chronic kidney disease (CKD) improves kidney injury by reducing fibrosis-related vascular calcification and declining apoptosis-linked nephron damage.

**Methods:**

The oral 25OHD-S was evaluated in 60,000 IU/month/36 weeks *versus* in 2000 IU/d/24 weeks in CKD Stage 3 with serum 25OHD level < 20 ng/mL. The study was undertaken on 156 black subjects and 150 white subjects Southern Sahara (SS). All biomarkers of cardiometabolic (CMet) and cardiorenal (CRenal) syndrome, Renin-angiotensin-aldosterone system (RAAS) profile, secondary hyperparathyroidism (SHPT), N-terminal pro B-type natriuretic peptide (NT-proBNP), Troponin T (cTnT) and atherogenicity risk were assessed by biochemical methods. Estimate glomerular filtration rate (eGFR) by chronic CKD-EPI equation formula. Total serum vitamin D by liquid chromatography-tandem mass spectrometry (MS).

**Results:**

Vitamin D deficiency alters in the same manner CMet, CRenal, and others biomarkers in both groups SS; however, these disorders are more acute in blacks compared to whites SS. Oral 25OHD-S a highlighted improvement of eGFR drop, SHPT decrease, decline proteinuria, and cardiac failure risk (NT-proBNP and cTnT) attenuation. Concomitantly, 25OHD-S normalizes *Renin*, *Aldosterone*, and *Angiotensin System* (RAAS) activity. Nevertheless, homocysteine and Lp (a) do not modulate by 25OHD-S.

**Conclusions:**

The oral vitamin D3 supplementation, according the dose, and the treatment duration does not like in black-skinned people *versus* to white-skinned inhabitants, while the 02 groups are native to the same Saharan environment. It emerge that a high intermittent dose through an extensive supplementation (60,000 IU/36 weeks) was more effective in black subjects. At opposite, a lower dose during a short period supplementation is sufficient (2000 IU/24 weeks) in white subjects.

## Introduction

The southern Sahara (SS) residents include both black and white subjects characterized by essential arterial hypertension (EAH) which is the major risk factor for chronic kidney disease (CKD). In the SS population, EAH represents 50.2% and CKD 5.8% (3.5 million cases) [1]. Recently, an epidemiological study has shown a strongly link between cardiometabolic (CMet) risk or CMet syndrome and race/ethnicity factor in kidney diseases [2]. CMet is characterized by insulin resistance, accretion of visceral adiposity, blood pressure (BP) disturbs, or hypertension, dyslipidemia, glucose intolerance or type 2 diabetes, including microalbuminuria to reduce renal function [3]. The *National Health and Nutrition Examination Survey* (NHANES) age-adjusted CMet prevalence is 31.1% in black and 9.6% in white Americans population [4]. Besides, some studies have shown the early association between CMet and Cardiorenal syndrome (CRenal) in CKD [5,6]. The CRenal affects chronic damage kidney and can lead to heart chronic dysfunction through excessive activation of the *Rennin-angiotensin-aldosterone system* (RAAS) [7]. The recent meta-analysis have demonstrated that CKD deterioration is related to alteration of bone and mineral metabolism as novel risk factors emerged for cardiovascular disease mortality in patients with CKD and CRenal. This relationship is explained by strongly linked with hyperphosphatemia and vascular calcification [8]. The RAAS play a central factor contributing to *end-stage kidney disease* (ESKD), particularly in black individuals [9]. According to several studies, *N-terminal pro B-type natriuretic peptide* (NT-proBNP) and Troponin T(cTnT) are the main cardio-renal axis biomarkers linked to CRenal which are implicated in decline of *estimated glomerular filtration rate* (eGFR) [10]. On the over hand, several studies have shown that CKD progression is strongly associated to vitamin D (25OH D) deficiency in general population [11], but more pronounced in black population than in white’s people [12]. The 25OH D deficiency can to lead some deleterious complications, mainly hypersecretion of parathyroid hormone (PTH) and secondary hyperparathyroidism (SHPT) [13]. Furthermore, the 25OH D deficiency affect the renal α-hydroxylase inhibition leads to depletion of 1,25-dihydroxyvitamin D [1,25(OH)2D] and an accumulation of *Fibroblast Growth Factor-23* (FGF-23) leads to hyperphosphatemia [14]. Likewise, some studies reveal that 25OH D deficiency in black’s people has higher serum phosphate and lower urinary phosphate excretion despite higher levels of FGF-23 and PTH [15]. The mineral disorders affect calcium metabolism and may contribute the SHPT severity to increased bone mass [16]. Some meta-analysis showed that active vitamin D (1,25(OH)2D3) exerts a beneficial renal protection in CKD patients by reducing proteinuria and inhibits vascular calcification fibrosis and nephrotic apoptosis [17]. It is for this reason that *Kidney Disease Improving Global Outcome* (KDIGO) consensus recommends routine prescription of calcitriol (1,25(OH)2D3) or analogues in adult patients from CKD3 according severe or mild vitamin D deficiency [18]. However, *National Kidney Foundation − Kidney Disease Outcomes Quality Initiative* (NKF-KDOQI) studies are controversial about vitamin D supplementation and not established consensus which defines the optimal level of vitamin D treatment to improve the glomerular filtration rate [19].

It is well established the protective effect of vitamin D3 in CKD patients (general population and Black American-African); however, no study was conducted in the black Maghreb population (Algeria, Tunisia, Morocco). The originality of this study is to compare white and black subjects living in a very sunny environment, yet the vitamin D deficiency is more marked in Blacks than in Whites. The genetic factor probably plays an important role, which is considered as a limiting factor in our study. In this study, we have tested the dose-response of 25-hydroyxvitamin D (Cholecalciferol) supplementation in continuously at low multi-doses (2000 IU/d) *versus* an intermittent at high dose (60,000 IU/month) during two periods (24 weeks *versus* 36 weeks) in CKD 3 adult’s black SS participants *versus* white SS participants with vitamin D deficiency. The objectives of this study are as follows: (i) correction vitamin D deficiency by increasing total serum vitamin D levels; (ii) inhibition to decline eGFR progression; (iii) Reduction PTH serum levels; (iv) Attenuation CMet and CRenal disorders; (v) Decrease atherogenicity risk. To our knowledge, this study is the first investigation according racial/ethnic disparities in the Algerian Saharan population.

## Materials and methods

It is important to specify that the cohort of this study is the same one that was used for our previous investigation in Black *versus* Whites SS population, which confirms the similarity of the enrollment participants and methods/measurements [20].

### Participants and study design

This study was a randomized, multicenter, controlled trial in CKD community-based SS Algerian regions. The Whites (W) and Blacks (B) participants were recruited from 2015 to 2020. In this study, we excluded participants CKD with eGFR > 90 and < 30 mL/min/1.73 m^2^, ESKD and dialysis; transplant patients; kidney cancer such concurrent active malignancies (malignant tubulo-interstitials) and patients with viral infection (hepatitis B and hepatitis C). We have also excluded participants under vitamin D supplementation (before this study), parathyroid and thyroid diseases and sarcoidosis. The starting cohort made up of 760 people who agreed to participate in this study. We only retained CKD3 and excluded CKD 1–2–4 stages. CKD Stage 3 was chosen on the basis of clinical and biological signs of vitamin D deficiency effects witch appear in Stage 3 (strongly increased PTH), but not in Stages 1 and 2. Stage 4 is a more intricate stage, particularly with bone complications (decrease bone mineral density). Indeed, the hypothesis of this investigation is to know whether vitamin D supplementation could prevent the deterioration of renal function from Stage 3, particularly with SHPT and bone resorption. If we had also considered Stage 4, the interpretation of the data would be more complex and the conclusion would be less relevant. Moreover, several studies describe this situation [21]. All participants were admitted to the clinical nephrology exploration, dialysis and kidney transplant unit according to skin color including white and black SS participants. We performed the baseline 25OHD assay in 156 white and 150 black SS participants with mild vitamin D deficiency (25OHD <50 nmol/L or <20 ng/mL). Participants started to receive vitamin D supplementation for the first time. Before vitamin D supplementation, participants completed by socio-demographic and dietary-physical activity questionnaires. To estimate food consumption pattern, a frequency questionnaire and 24-h recall were applied to study participants. The method is described in the TAHINA-Maghreb study (Transition and health impact in North Africa) [22] according to 139 questions on 124 foodstuffs. The food intake, such as vitamin D, calcium, proteins, sodium, and drinking water were evaluated according to the CIQUAL table. The drug doses were stable throughout this study.

### Informed consent statement

This Renal study protocol (*Algiers Ethnic-Renal Study*) was approved by the *Ethics Committee of Algerian Ministry of Public Health* (ECAMPH) and conformed to the principles outlined in the declaration of Helsinki (http://www.wma.net). Ethical approval code: The permits and ethical rules have been achieved according to the Executive Decree no. 10–90 (10 March 2010) completing the Executive Decree no. 04–82 (18 March 2004) of the Algerian Government, establishing the terms and approval modalities.

### Clinical vitamin D3 supplementation protocol study

**Figure 1.**
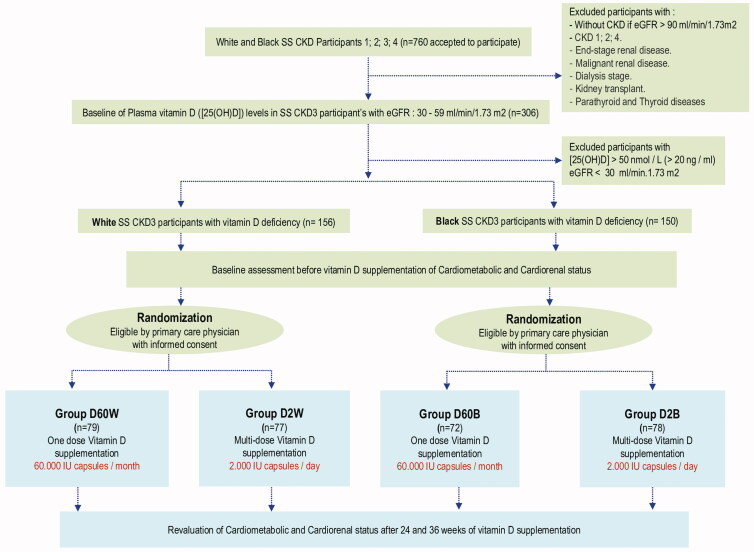
Clinical protocol randomization algorithm of vitamin D3 supplementation in CKD Stage 3 Southern Sahara (SS) White (W) and Black (B) people at 2000 IU doses every day (D2 group) *versus* 60,000 IU doses every month (D60 group) during 24 and 36 weeks vitamin D3 treatment.

We adopted the NKF guidelines that maintain sufficient levels of 25(OH) D are ≥30 ng/mL (>75 nmol/L). We proceeded to the cohort randomization (Figure 1) into 4 stratified groups depending on the dose of vitamin D to supplement: i) 77 W and 78 B participants received multi-dose of 25-hydroyxvitamin D (Cholecalciferol) supplementation of 2000 IU/mL capsules every day; ii) 79 W and 72 B participants received a single dose of 60,000 IU/mL of Cholecalciferol capsules every month. Cholecalciferol was dosed at 2000 IU comes from the Italfarmaco S.A. laboratories, Industrial de Alcobendas, Madrid, Spain. Cholecalciferol dosed at 60,000 IU comes from the Sanofi laboratories Product leaflet of DePURA, India. Regarding 60,000 IU vitamin D3 supplementation, black and white SS CKD participants were supplemented in the beginning with 60,000 IU weekly for 4 weeks, then 60,000 IU for 24 weeks and 36 weeks monthly. CMet and CRenal biomarkers have been revaluated after 24 and 36 weeks vitamin D supplementation. All clinical explorations participants have been examined by the same physician.

### CKD screening

The CKD was explored followed bio-clinical criteria according the KDIGO guidelines. The CKD stage was evaluated by eGFR using the chronic CKD-EPI equation formula (CKD *– EPIdemiology collaboration*) [23]. CKD is usually defined from a KDIGO by eGFR <60 mL/min per 1.73 m^2^ [24]. KDIGO proposes that the calculation of eGFR equation in adults CKD should take into account the racial-ethnic factor (black *versus* white) and sex [25].

In this study, participants are classified as CKD3 based on eGFR of 30 − 59 mL/min/1.73 m^2^; subdivided into Stage 3 A (45 − 59 mL/min/1.73 m^2^) and Stage 3B (30 − 44 mL/min/1.73 m^2^). Proteinuria was assessed by 24-h urine collection. Albuminuria was categorized as microalbuminuria defined by a urine aAlbumin-to-creatinine ratio (uACR) ≥30 µg/mg or >30 mg/24 h. The uACR normal range is between 0.34 and 3.39 mg/mmol/24 H and macroalbuminuria if uACR >3.39 mg/mmol/24H according to previous studies [26].

### Cadiometabolic risk screening

The cadiometabolic risk is identified as the c CMet syndrome. The CMet was diagnosed according to the *National Cholesterol Education Program Third Adult Treatment Panel* (NCEP/ATPIII) [27]. Insulin resistance was calculated by the homeostasis model assessment insulin resistance (HOMA-IR) method [28]. Percent body fat (BF%) was calculated using the formula validated by Deurenberg: BF% = (1.2 × body mass index (BMI) + (0.23 × age) − (10.8 × S) − 5.4 (S is the gender correction factor) [29]. However, recent evidence has shown the usefulness of bioelectrical impedance analysis which is an easy and noninvasive method to better focus the various parameters of body composition [30].

### Cadiorenal syndrome screening

The Cadiorenal syndrome (CRrenal) was diagnosed according to the recommendations of the NKF Consensus [31]. In this study, the specific biomarkers CRrenal were evaluated by NT-proBNP, high-sensitivity cardiac troponin T (*hs-cTnT*) and RAAS.

### Atherogenicity risk

The Atherothromboembolic biomarkers have been added in this study such as lipoprotein (a), homocysteine (hCys) and Apo B_100 −_ Apo A_1_ ratio.

### Blood samples analysis methods

All participants were admitted to the hospital at 7 am after 12 h of fasting before medication. Blood samples from participants were centrifuged at 3000 rpm for 10 min. Serum or plasma samples were immediately put on ice and kept frozen at −80 °C until analyses were performed. Fasting glucose, triglycerides (TG), total cholesterol (TC), high-density lipoprotein cholesterol (HDL-C), phosphorus, calcium, uric acid, urea, albumin, and total proteins were determined by enzymatic methods using an automatic biochemical analyzer (Roche Diagnostics, Meylan, France). Microalbuminuria was assessed by immunoturbidimetry. The low-density lipoprotein cholesterol (LDL-C) was calculated using Friedewald’s formula applied to subjects with CMet. Plasma sodium, potassium, and phosphorus levels were measured by flame photometry method. Plasma hCys were determined by *Fluorescence Polarization Immuno-Assays* (FPIA). Plasma insulin was measured using a double-antibody solid phase radioimmunoassay. Plasma total intact PTH was measured by an electro-chemiluminescence immunoassay run. Based on the KDIGO recommendations [32]; Hyper-parathyroidism was defined as PTH >65 pg/mL or >6.89 pmol/L. Plasma Aldosterone levels, Plasma Angiotensin II levels and Plasma Renin activity were determined by ELISA assay kit (Isbio, Seattle, WA). Plasma NT-proBNP levels and hs-cTnT were measured with a sandwich chemiluminescence immunoassay (Modular Analytics E170 Roche Diagnostics, Meylan, France).

### Urine Collection and 24-h UCr excretion measurements

All participants are requested to provide at least one 24-h urine collection. Fasting urinary Creatinine (UCr) was assayed by a spectrophotometric reaction according to the Jaffe method (Roche Diagnostics, Indianapolis, IN). UCr was calculated as the product of urinary creatinine levels and 24-h urinary volume. Microalbuminuria was determined by immunoturbidimetry. Urinary calcium and phosphorus were assayed on colorimetric method (Abbott Diagnostics, Irving, TX). Fasting urinary uric acid was determined by a kinetic uricase method. Urinary Sodium and Potassium excretion were estimated by Kawasaki formula considering the dietary sodium and potassium intake [33].

### Serum 25-OH Vitamin D and 1, 25(OH) 2 D assessment

Vitamin D status was measured as serum 25(OH) D concentrations (including both D2 and D3 isomers) were performed using a high-performance liquid chromatography (HPLC) method coupled with mass spectrometry (MS) [34]. The range of the vitamin D assay is 2.4 − 114 ng/mL or 6 − 285 nmol/L. Serum 1,25(OH)2D was measured by the method of Reinhardt et al. [35]. The range of the 1, 25(OH) 2 D assay is 18 − 78 pg/mL or 43.2 − 187 pmol/L.

### Physical activity evaluation

The physical activity level was assessed using the self-administered International Physical Activity Questionnaire (IPAQ) [36]. The participants were classified into four categories: (i) no regular physical activity with a sedentary lifestyle; (ii) minimal physical activity (<75 min/weeks); (iii) insufficient physical activity (≥75 min < 150 min/weeks); (iv) and sufficient physical activity (≥150 min/weeks).

### Statistical analysis

All statistical analyses were performed with Epi-info version 5 and Statview version 5 (Abacus Concepts, Berkeley, CA). Data (normally distributed) are presented as mean ± standard deviation (SD). Student’s *t*-test and one-way ANOVA were used for the comparison between the CKD Black *versus* CKD White groups for the same dose of vitamin D supplementation: D2W *versus* D2B and D60W *versus* D60B, mainly to analyze the relationship between vitamin D deficiency in CKD Black and White groups and severity of CVD in both groups, essentially NT-proBNP and RAAS biomarkers. Pearson’s correlation analysis was performed to quantify associations between the each dose effects of vitamin D supplementation and CMet or CRenal clusters, eGRF, PTH, and atherogenicity biomarkers. The results were considered significant at *p < 0.05, very significant at **p < 0.01, or highly significant at ***p < 0.001.

## Results

This study is based on multi-analysis biomarkers interactions and correlated with the vitamin D3 supplementation (25OHD-S) effects at two doses and two treatment times according the racial/ethnicity factor. Our data have been grouped in status form: metabolic, renal, hormonal, and atherogenic.

### Clinical cohort characterization

The description of clinical SS population by sex and race/ethnicity is detailed in Table 1. In reference to skin color, we listed more black SS than white SS with no age or gender difference. In this investigation, CKD affects significantly (*p* < 0.001) more black SS than their white SS people. It is important to note that obesity, essential hypertension, type 2 diabetes, and hypocalcemia are the most dominant pathologies associated with CKD in the Black SS group *versus* the White SS group (respectively, 60.5, 60, and 42.8%, *p* < 0.001). Hyperuricemia and heart failure are other pathologies linked to CKD in the black SS group, but less accented compared to the White SS group. Nevertheless, coronary artery failure, vascular diseases, lipid disorders, and anemia were more recurrent comorbidities in white SS than in black SS (*p* < 0.01). We did not unregistered difference for the tobacco and alcohol consumption. The therapeutic protocol is the same for the 02 black SS and White SS groups. Unpredictably, the black SS group seemed to consume more vitamin D (present in food) than the White SS group, while both groups were vitamin D deficient (*p* < 0.001). Additionally, sodium and food calorie intakes are significantly elevated (*p* < 0.001) in the black SS group *versus* White SS group. Conversely, calcium intake is significantly higher (*p* < 0.001) in the White SS group *versus* black SS group. Compared to the white SS participants, black SS counterpart is largely a sedentary lifestyle.

**Table 1.** Baseline clinical characteristics in Blacks *versus* Whites SS in general population.

Parameters	Whites (*N* = 79)	Blacks (*N* = 72)	*p* Value baseline
Skin color (%)	46.3	53.7	<0.01
Age (year)	52 ± 3	49 ± 1	0.072
Gender (%)	48/33 (F/M)	53/41 (F/M)0.083	
CKD3 (%)	37	49	<0.001
Comorbidities (%)			
Hypertension	60.5	74.9	<0.001
Diabetes	26.7	32.6	<0.001
Anemia	66.6	57.2	<0.01
Coronary artery disease	45	32	<0.01
Congestive heart failure	19	25	<0.01
Cerebral vascular accident	15	9	<0.01
Peripheral vascular disease	19	12	<0.01
Obesity	60	86	<0.001
Dyslipidemia	75	28	<0.01
Hypocalcemia	42.8	60.6	<0.01
Hyperuricemia	22.5	36.5	<0.01
Smoking current (%)	20	18	0.091
Alcohol use (%)	2	1	0.073
Current drug use (%)			
Calcium channel blocker	45	52	0.144
β-Blockers	45	46	0.061
Thiazide	7	10	0.205
Diuretic	63	76	0.331
Aspirin	56	58	0.069
Statins	28	39	0.177
Vitamin D intake (µg/d)	69 ± 4	97 ± 3	<0.001
Vitamin D intake (IU/d)	2760 ± 160	3880 ± 120	<0.001
Calcium intake (mg/d)	977 ± 11	691 ± 22	<0.01
Protein intake (g/kg/d)	0.55 ± 0.03	0.99 ± 0.07	0.366
Drinking water intake (L/d)	1.45 ± 0.05	1.42 ± 0.08	0.063
Sodium intake (g/d)	2.53 ± 0.19	5.64 ± 0.21	<0.001
Caloric intake (kcal/kg/d)	35 ± 5	62 ± 9	<0.001
Physical activity (min/week)	60 ± 5	40 ± 3	<0.01

F: female; M: male; CKD3: chronic kidney disease stage 3; SS: Southern Sahara; *p*: significance degree.

Data are reported as mean ± SD (standard deviation) or proportions. Data of Vitamin D and Calcium intake are given before oral Vitamin D supplementation. One microgram of vitamin D is equal to 40 IU.

### Effects of oral 25OHD-S on 25-(OH) D and 1, 25-(OH) 2 D serum levels

The data unregistered in Tables 2 and 3 illustrate a severe 25-OHD deficiency in the blacks SS group compared to the whites SS group. At opposite, SS blacks manifested significantly more 2 D 1,25(OH) serum compared to SS whites. A positive correlation was found between 25-OHD deficiency and lower eGFR in both groups. This difference is more pronounced in the black SS group compared to the White SS group (*r* = +0.79, *p* < 0.001; *r* = +0.46, *p* < 0.01, respectively). Clinically, 25OHD-S was well tolerated by all participants. No serious adverse effects were observed. Additionally, no participant in either group developed hypercalcemia. The data mentioned in Tables 2 and 3 characterize the dose-response effect between 25OHD-S and CMet clusters in participants of the 02 groups. Dose-dependent oral 25OHD supplementation significantly increased serum 25(OH)D levels and 1,25(OH)2D levels in both SS groups compared to baseline levels. Vitamin D treatment was immediate and sustained in the White SS group from 24 weeks. However, in the Black SS group, it was observed slowly and up to 36 weeks. The beneficial effect was obtained at 2000 IU (D2) and 60,000 IU (D60), concomitantly at 24 and 36 weeks of 25OHD-S. Unexpectedly, serum 25OHD levels were not normalized at dose D2 in Black SS group both at 24 and at 36 weeks compared to White SS group and persisted in the deficient state (vitamin D3 < 30 ng/mL). Even so, Black SS groups achieved treatment target by 25OHD-S (vitamin D3 > 30 ng/mL) at D60-36 weeks according increase of 1.25 (OH) 2 D by 84% and 53%, respectively (*p* < 0.001).

**Table 2.** Effects of oral 2000 IU vitamin D3 supplementation/d at 24 and 36 weeks on the metabolic syndrome clusters and atherothrombogenic biomarkers in Blacks *versus* Whites SS CKD Stage 3 participants.

	Baseline		24 Weeks		36 Weeks		
Parameters	WSS (*N* = 79)	BSS (*N* = 72)	WSS (*N* = 74)	BSS (*N* = 70)	WSS (*N* = 71)	BSS (*N* = 68)	*p* Value
25(OH)D (ng/mL)	19.8 ± 4.33	6.77 ± 1.51	30.9 ± 5.18	19.1 ± 6.15	48.1 ± 4.22	26.8 ± 3.54	<0.001
1,25(OH)2D (pg/mL)	29.9 ± 7.51	44.3 ± 3.55	40.4 ± 1.66	72.6 ± 2.89	55.3 ± 3.11	87.9 ± 1.98	<0.001
BMI (kg/m^2^)	28.1 ± 4.25	32.3 ± 1.26	27.2 ± 2.22	31.8 ± 2.63	26.6 ± 1.15	30.2 ± 1.99	<0.01
WC (cm)	84.9 ± 3.98	107 ± 6.90	83.9 ± 5.55	100 ± 3.89	82.5 ± 4.11	101 ± 2.11	<0.001
WC/WH ratio	1.09 ± 0.02	1.13 ± 0.02	1.05 ± 0.01	1.11 ± 0.04	1.03 ± 0.03	1.09 ± 0.05	<0.01
BF (%)	46.6 ± 2.17	55.1 ± 8.12	45.9 ± 3.32	54.5 ± 4.11	39.3 ± 7.71	53.9 ± 2.41	<0.001
Glycemia (mmol/L)	6.70 ± 1.49	5.91 ± 1.97	6.38 ± 1.09	5.66 ± 1.83	5.67 ± 1.11	5.49 ± 1.33	0.223
Insulinemia (pmol/mL)	81 ± 7.15	114 ± 2.91	77.7 ± 3.55	105 ± 5.62	72.9 ± 5.76	95.6 ± 4.79	<0.001
HOMA-IR	3.50 ± 0.93	5.01 ± 0.67	3.17 ± 0.11	4.50 ± 0.55	2.64 ± 0.34	4.09 ± 0.26	<0.001
Triglycerides (mmol/L)	2.19 ± 0.28	1.45 ± 0.26	1.84 ± 0.71	1.32 ± 0.31	1.65 ± 0.05	1.15 ± 0.01	<0.001
Total cholesterol (mmol/L)	5.84 ± 0.68	4.75 ± 0.32	5.39 ± 0.88	4.26 ± 0.43	5.12 ± 0.33	3.82 ± 0.81	<0.001
HDL-C (mmol/L)	0.85 ± 0.08^(M)^	0.95 ± 0.18^(M)^	1.14 ± 0.3^(M)^	1.09 ± 0.17^(M)^	1.24 ± 0.04^(M)^	1.25 ± 0.04^(M)^	<0.01
	1.11 ± 0.15^(F)^	1.24 ± 0.13^(F)^	1.21 ± 0.8^(F)^	1.27 ± 0.05^(F)^	1.26 ± 0.03^(F)^	1.28 ± 0.02^(F)^	<0.01
LDL-C (mmol/L)	4.52 ± 0.81	3.17 ± 0.79	4.29 ± 0.27	3.02 ± 0.44	4.06 ± 0.16	2.66 ± 0.23	<0.001
SBP (mm Hg)	141 ± 3	159 ± 7	135 ± 1	157 ± 2	130 ± 5	155 ± 6	<0.01
DBP (mm Hg)	72 ± 3	90 ± 2	70 ± 5	87 ± 1	68 ± 3	85 ± 2	0.145
Apoprotein A_1_ (µmol/L)	42.4 ± 2.17	41.1 ± 3.66	44.1 ± 2.09	43.7 ± 3.11	46.9 ± 7.88	45.3 ± 3.77	<0.02
Apoprotein B_100_ (µmol/L)	35.9 ± 5.83	38.3 ± 7.15	33.4 ± 1.77	36.4 ± 4.51	32.9 ± 2.24	33.1 ± 1.91	<0.01
Apo B_100_ −Apo A_1_ ratio	0.846 ± 0.06	0.931 ± 0.07	0.757 ± 0.03	0.832 ± 0.05	0.702 ± 0.07	0.717 ± 0.04	<0.01
Lp (a) (nmol/L)	78.5 ± 4.10	94.5 ± 3.27	76.9 ± 6.11	92.8 ± 1.72	72.1 ± 2.66	91.2 ± 3.27	0.772
tHcy (µmol/L)	17.2 ± 2.52	21.1 ± 3.31	16.5 ± 2.15	19.8 ± 2.81	16.9 ± 3.55	20.7 ± 1.88	0.568

CKD3: chronic kidney disease Stage 3; SS: Southern Sahara; F: female; M: male; 25(OH)D: total serum 25 hydroxyvitamin D; 1,25(OH)2D: 1,25-dihydroxyvitamin D; BMI: body mass index; WC: waist circumference; WH: waist hips; BF: percent body fat; HOMA: Homeostasis Model Assessment; C: cholesterol; HDL: high-density lipoprotein; LDL: low-density lipoprotein; SBP: systolic blood pressure; DBP: diastolic blood pressure; Lp (a): lipoprotein (a); tHcy: total homocysteine; ApoB100/ApoA_1_: Apolipoprotein B100/Apolipoprotein A.

The mean values are assigned from the standard error to the mean (X ± ESM). The degree of significance is calculated for a risk of error *α* = 5%. The mean comparison is established for each group Whites SS and Blacks SS. Baseline data were obtained before vitamin D supplementation. *p* Value is calculated at baseline time (Whites SS *versus* Blacks SS groups).

**Table 3.** Effects of oral 60,000 IU vitamin D3 supplementation/month at 24 and 36 weeks on the metabolic syndrome clusters and atherothrombogenic biomarkers in CKD Stage 3 Blacks *versus* Whites SS participants.

	Baseline		24 weeks		36 weeks		
Parameters	WSS (*N* = 79)	BSS (*N* = 72)	WSS (*N* = 74)	BSS (*N* = 70)	WSS (*N* = 71)	BSS (*N* = 68)	*P* value
25OHD (ng/mL)	19.8 ± 4.33	6.77 ± 1.51	31.5 ± 4.11	22.7 ± 5.09	46.3 ± 3.42	44.9 ± 2.45	<0.001
1,25(OH)2D (pg/mL)	29.9 ± 7.51	44.3 ± 3.55	41.2 ± 2.58	82.6 ± 3.77	56.2 ± 5.08	95.8 ± 2.09	<0.001
BMI (kg/m^2^)	28.1 ± 4.25	32.3 ± 1.26	27.2 ± 3.11	31.1 ± 1.37	26.7 ± 2.51	30.1 ± 2.08	<0.01
WC (cm)	84.9 ± 3.98	107 ± 6.90	83.4 ± 3.74	103 ± 2.98	82.5 ± 3.21	99.1 ± 3.77	<0.001
WC/WH ratio	1.09 ± 0.02	1.13 ± 0.02	1.05 ± 0.02	1.11 ± 0.03	1.02 ± 0.01	1.01 ± 0.02	<0.01
BF (%)	46.6 ± 2.17	55.1 ± 8.12	45.7 ± 2.23	53.7 ± 3.21	40.1 ± 5.18	52.1 ± 3.14	<0.001
Glycemia (mmol/L)	6.70 ± 1.49	5.91 ± 1.97	6.55 ± 1.17	5.46 ± 1.37	5.69 ± 1.71	5.38 ± 1.24	0.251
Insulinemia (pmol/mL)	81 ± 7.15	114 ± 2.91	73.5 ± 2.81	92.1 ± 4.26	71.5 ± 3.67	80.9 ± 3.92	<0.001
HOMA- IR	3.50 ± 0.93	5.01 ± 0.67	3.10 ± 0.14	3.80 ± 0.27	2.60 ± 0.43	3.30 ± 0.63	<0.001
Triglycerides (mmol/L)	2.19 ± 0.28	1.45 ± 0.26	1.80 ± 0.77	1.21 ± 0.13	1.68 ± 0.08	1.09 ± 0.02	<0.01
Total Cholesterol (mmol/L)	5.84 ± 0.68	4.75 ± 0.32	4.94 ± 0.77	4.31 ± 0.35	4.40 ± 0.22	3.91 ± 0.17	<0.001
HDL-C (mmol/L)	0.85 ± 0.08^(M)^	0.95 ± 0.18^(M)^	1.12 ± 0.01^(M)^	1.18 ± 0.11^(M)^	1.24 ± 0.04^(M)^	1.27 ± 0.05^(M)^	<0.01
	1.11 ± 0.15^(F)^	1.24 ± 0.13^(F)^	1.25 ± 0.05^(F)^	1.28 ± 0.05^(F)^	1.26 ± 0.03^(F)^	1.30 ± 0.04^(F)^	<0.01
LDL-C (mmol/L)	4.52 ± 0.81	3.17 ± 0.79	4.01 ± 0.73	3.08 ± 0.31	3.61 ± 0.74	2.63 ± 0.33	<0.001
SBP (mm Hg)	141 ± 3	159 ± 7	136 ± 3	150 ± 4	131 ± 4	147 ± 5	<0.01
DBP (mm Hg)	72 ± 3	90 ± 2	69 ± 2	85 ± 2	67 ± 1	83 ± 2	0.178
Apoprotein A_1_ (µmol/L)	42.4 ± 2.17	41.1 ± 3.66	43.8 ± 1.98	44.9 ± 2.72	45.1 ± 5.09	47.2 ± 3.54	<0.001
Apoprotein B_100_ (µmol/L)	35.9 ± 5.83	38.3 ± 7.15	32.7 ± 1.33	34.3 ± 2.17	31.7 ± 1.89	30.1 ± 1.74	<0.02
Apo B_100_- Apo A_1_ ratio	0.846 ± 0.06	0.931 ± 0.07	0.746 ± 0.02	0.763 ± 0.05	0.744 ± 0.05	0.637 ± 0.02	<0.01
Lp (a) (nmol/L)	78.5 ± 4.10	94.5 ± 3.27	77.5 ± 3.71	91.1 ± 1.22	76.9 ± 1.87	90.7 ± 2.37	0.401
tHcy (µmol/L)	17.2 ± 2.52	21.1 ± 3.31	16.1 ± 1.54	18.8 ± 1.27	15.8 ± 2.32	18.3 ± 1.09	0.711

CKD3: Chronic kidney disease Stage 3; SS: Southern Sahara; F: female; M: male; 25(OH)D: total serum 25 hydroxyvitamin D; 1,25(OH)2D: 1,25-dihydroxyvitamin D; BMI: body mass index; WC: waist circumference; WH: waist hips; BF: percent body fat; HOMA: Homeostasis Model Assessment; C: cholesterol; HDL: high-density lipoprotein; LDL: low-density lipoprotein; SBP: systolic blood pressure; DBP: diastolic blood pressure; Lp (a): lipoprotein (a); tHcy: total homocysteine; ApoB100/ApoA_1_: Apolipoprotein B100/Apolipoprotein A.

The mean values are assigned from the standard error to the mean (X ± ESM). The degree of significance is calculated for a risk of error *α* = 5%. The mean comparison is established for each group Whites SS and Blacks SS. Baseline data were obtained before vitamin D supplementation. *p* Value is calculated at baseline time (Whites SS *versus* Blacks SS groups).

### Effects of oral 25OHD-S on clusters of cardiometabolic syndrome

The compounds of CMet syndrome in SS participants according to race and ethnicity were described in Tables 2 and 3.

#### Anthropometric status

An association was observed between BMI and body fat percentage, characterized by subcutaneous-abdominal adipose tissue. We found that Black SS group is obese compared to white group. According to the NCEP-ATPIII definition, visceral adiposity is established by increased waist circumference (WC) in Black SS group compared to White SS group. The WC/Waist Hips (WH) ratio and the body fat accumulation confirm the android obesity phenotype in SS black men compared to the gynoid obesity phenotype in SS black women. BMI was significantly correlated with body fat percentage in blacks SS group (*r* = +0.49, *p* < 0.01) but not in whites SS group. 25OHD-S led to a moderate reduction in BMI, WC, WC/WH ratio, and body fat percentage in both groups. The 25OHD-S effects were cholecalciferol dose-dependent and the treatment duration. We found an insignificant attenuation in White SS group at D2-36 weeks (−6, −3, −6, and −15%, respectively). Moreover, anthropometric status was not significantly improved in the Black SS group at D60-36 weeks (−7, −7, −10, and −6%, respectively).

#### Glycemia and insulin resistance (Homa-IR) status

At baseline, the data presented in Tables 2 and 3 do not reveal hyperglycemia in both groups (glycemia <6.99 mmol/L or <1.26 g/L); however, black participants showed glucose intolerance than in whites SS group. It is important to note that carbohydrate intolerance is associated with hyperinsulinism in both groups. The insulin sensitivity disorder is significantly more pronounced in black SS participants than in white SS participants. A significant positive correlation was observed between Homa-IR and WC in Black SS group, but not in White SS group (*r* = +0.65, *p* < 0.001). On the contrary, we did not establish an association between Homa-IR and body fat percentage. The 25OHD-S data mentioned in Tables 2 and 3 reveal a significant reduction in fasting glycemia, insulinemia, and Homa-IR at D2-36 weeks in white SS participants (−14, 10, and −24%, respectively, *p* < 0.05) and at D60-36 weeks in black SS counterpart (−9, −29, −34%, respectively, *p* < 0.01).

#### Dyslipidemia profile

In baseline, data exhibited in Tables 2 and 3 show that lipid disturb is only present in white SS participants but absent in black SS participants. Cholesterolemia and triglyceridemia were significantly lesser in blacks SS group than in White SS group. Similarly, serum LDL cholesterol concentrations were also significantly lower in Black SS group *versus* White SS group. In contrast, serum HDL-cholesterol levels were significantly higher in Black SS group than in White SS group. In addition, we noticed that serum HDL levels were higher in black SS women than in white SS women. 25OHD-S decreased significantly serum TG and cholesterol at D2-36 weeks in white SS group (−20, −19%, respectively) and at D60-36 weeks in Black SS group (−24, −17, respectively, *p* < 0.02). Conversely, 25OHD-S enhances HDL cholesterol levels in both groups. This increase is significant at D2-36 weeks in white SS group (+24%, respectively, *p* < 0.01) and significantly reduced at D60-36 weeks in Black SS group (+25%, respectively, *p* < 0.01).

#### Blood pressure status

Baseline data recorded in Tables 2 and 3 illustrate that despite a normalized lipid profile, the Black SS group showed higher systolic blood pressure (SBP) compared to White SS group (*p* < 0.001). 25OHD supplementation influences BP through an inhibitory effect. 25OHD-S exerts a slight decrease SBP in White SS group at D2-36 weeks. At opposite, 25OHD-S does not normalize the SBP disorder in Black SS group (>140 mm Hg) while DBP is normal.

### Effects of oral 25OHD-S on the biomarkers of cardiorenal syndrome

The CRenal parameters data are potted in Tables 4 and 5. In this investigation, the black and white SS subjects were advanced CKD (Stage 3).

**Table 4.** Effects of oral 2000 IU vitamin D3 supplementation/day at 24 and 36 weeks on serum vitamin D level and renal biomarkers in Blacks *versus* Whites SS CKD Stage 3 participants.

	Baseline		24 Weeks		36 Weeks		
Parameters	WSS (*N* = 79)	BSS (*N* = 72)	WSS (*N* = 74)	BSS (*N* = 70)	WSS (*N* = 71)	BSS (*N* = 68)	*p* Value
eGFR (mL/min per 1.73 m^2^)	48.2 ± 2.31	45.4 ± 2.29	52.2 ± 9.09	49.6 ± 7.11	61.6 ± 4.22	55.8 ± 3.11	<0.001
S-Creatinine (µmol/L)	168 ± 19	221 ± 27	146 ± 63	199 ± 18	130 ± 13	161 ± 22	<0.001
U-Creatinine (mmol/24 h)	10.9 ± 2.11	14.8 ± 2.17	13.7 ± 1.99	18.5 ± 3.34	17.6 ± 1.09	22.8 ± 1.77	<0.001
S-Creatinine – BMI ratio	5.97 ± 1.22	6.84 ± 1.31	5.52 ± 1.11	6.25 ± 1.43	5.01 ± 1.31	5.99 ± 1.18	<0.001
S-Uric acid (µmol/L)	404 ± 36	441 ± 22	359 ± 17	410 ± 20	287 ± 32	366 ± 17	<0.001
U-Uric acid (mmol/24 h)	2.45 ± 0.66	2.71 ± 0.45	2.67 ± 0.19	2.97 ± 0.31	2.91 ± 0.24	3.21 ± 0.55	<0.001
S-Ca (mmol/L)	2.32 ± 0.62	2.20 ± 0.33	2.39 ± 0.61	2.44 ± 0.39	2.51 ± 0.16	2.65 ± 0.71	<0.02
U-Ca(mmol/24 h)	41.4 ± 3.41	25.2 ± 4.33	47.9 ± 6.11	29.7 ± 2.41	52.4 ± 1.99	32.4 ± 3.11	<0.02
iCa (mmol/24 h)	1.17 ± 0.15	1.30 ± 0.25	1.20 ± 0.19	1.41 ± 0.55	1.38 ± 0.11	1.49 ± 0.31	<0.02
S-Alb (µmol/L)	595 ± 44	580 ± 33	665 ± 71	609 ± 55	744 ± 24	720 ± 19	<0.001
U-Alb (mg/24 h)	40.5 ± 7.11	54.8 ± 3.27	31.9 ± 2.08	42.5 ± 1.44	29.7 ± 3.11	34.9 ± 2.71	<0.001
UACR (mg/mmol/24 h)	5.71 ± 0.32	4.35 ± 0.44	4.32 ± 0.11	3.29 ± 0.73	3.68 ± 0.54	2.53 ± 0.66	<0.001
S-Sodium (mmol/L)	139 ± 3	147 ± 1	137 ± 7	142 ± 3	136 ± 6	143 ± 2	0.038
U-Sodium (mmol/24 h)	126 ± 2	123 ± 3	138 ± 4	135 ± 2	153 ± 7	150 ± 5	<0.01
S-Potassium (mmol/L)	4.33 ± 0.69	4.10 ± 0.55	4.27 ± 0.57	4.07 ± 0.19	4.24 ± 0.33	4.05 ± 0.75	0.045
U-Potassium (mmol/24 h)	63 ± 3	42 ± 5	69 ± 4	47 ± 2	73 ± 5	54 ± 3	<0.01
S-Phosphorus (mmol/L)	1.20 ± 0.19	1.19 ± 0.11	1.15 ± 0.81	1.16 ± 0.44	1.12 ± 0.73	1.14 ± 0.55	0.072
U-Phosphorus (mmol/24 h)	302 ± 25	221 ± 17	272 ± 51	211 ± 33	254 ± 13	209 ± 47	<0.01

SS: Southern Sahara; eGFR: estimated Glomerular filtration rate; S: Serum; U: urinary; S-Ca: serum calcium; U-Ca: urinary calcium; iCa: ionized Calcium; S-Alb: serum albumin; UACR: urinary albumin-creatinine ratio; U-Alb: Micro albuminuria.

The mean values are assigned from the standard error to the mean (X ± ESM). The degree of significance is calculated for a risk of error *α* = 5%. The mean comparison is established for each group Whites SS and Blacks SS. Baseline data were obtained before vitamin D supplementation. *p* Value is calculated at baseline time (Whites SS *versus* Blacks SS groups).

**Table 5.** Effects of oral 60,000 IU vitamin D3 supplementation/month at 24 and 36 weeks on serum vitamin D level and renal biomarkers in Blacks *versus* Whites SS CKD Stage 3 participants.

	Baseline		24 Weeks		36 Weeks		
Parameters	WSS (*N* = 79)	BSS (*N* = 72)	WSS (*N* = 74)	BSS (*N* = 70)	WSS (*N* = 71)	BSS (*N* = 68)	*p* Value
eGFR (mL/min per 1.73 m^2^)	48.2 ± 2.31	45.4 ± 2.29	49.7 ± 5.32	55.1 ± 5.22	58.7 ± 4.11	61.9 ± 2.72	<0.001
S-Creatinine (µmol/L)	168 ± 19	221 ± 27	154 ± 44	178 ± 22	137 ± 25	144 ± 13	<0.001
U-Creatinine (mmol/L)	10.9 ± 2.11	14.8 ± 2.17	13.1 ± 2.08	20.5 ± 1.88	16.7 ± 3.11	25.1 ± 2.09	<0.001
S-Creatinine-BMI ratio	5.97 ± 1.22	6.84 ± 1.71	5.25 ± 2.33	5.57 ± 2.07	4.76 ± 2.45	5.34 ± 1.87	<0.001
S-Uric acid (µmol/L)	404 ± 36	441 ± 22	360 ± 22	374 ± 31	289 ± 24	311 ± 55	<0.001
U-Uric acid (mmol/24 h)	2.45 ± 0.66	2.71 ± 0.45	2.62 ± 0.88	3.05 ± 0.14	2.95 ± 0.43	3.49 ± 0.27	<0.001
S-Ca (mmol/L)	2.32 ± 0.62	2.20 ± 0.33	2.40 ± 0.17	2.66 ± 0.88	2.55 ± 0.27	2.77 ± 0.19	<0.02
U-Ca (mmol/24 h)	41.4 ± 3.41	25.2 ± 4.33	48.7 ± 5.09	33.5 ± 1.18	53.5 ± 2.23	38.9 ± 3.51	<0.02
iCa (mmol/L)	1.17 ± 0.15	1.30 ± 0.25	1.19 ± 0.22	1.43 ± 0.19	1.39 ± 0.31	1.52 ± 0.15	<0.02
S-Alb (µmol/L)	595 ± 44	580 ± 33	632 ± 55	649 ± 23	747 ± 31	785 ± 27	<0.001
U-Alb (mg/24 h)	40.5 ± 7.11	54.8 ± 3.27	31.2 ± 1.31	37.8 ± 2.09	28.3 ± 4.07	31.4 ± 3.18	<0.001
S-Sodium (mmol/L)	139 ± 3	147 ± 1	138 ± 4	143 ± 2	136 ± 7	142 ± 3	0.062
U-Sodium (mmol/24 h)	126 ± 2	123 ± 3	139 ± 7	144 ± 4	151 ± 6	158 ± 5	<0.01
S-Potassium (mmol/L)	4.33 ± 0.69	4.10 ± 0.55	4.30 ± 0.25	4.08 ± 0.19	4.27 ± 0.17	4.01 ± 0.43	0.049
U-Potassium (mmol/24 h)	63 ± 3	42 ± 5	68 ± 6	49 ± 4	72 ± 3	50 ± 1	<0.01
U-Sodium-to-Potassium ratio	2.01 ± 0.11	2.92 ± 0.23	2.04 ± 0.15	2.93 ± 0.41	2.09 ± 0.17	3.16 ± 0.33	<0.001
S-Phosphorus (mmol/L)	1.20 ± 0.19	1.19 ± 0.11	1.14 ± 0.21	1.15 ± 0.16	1.11 ± 0.83	1.12 ± 0.71	0.077
U-Phosphorus (mmol/24 h)	302 ± 25	221 ± 17	248 ± 17	207 ± 21	222 ± 18	205 ± 55	<0.01

SS: Southern Sahara; eGFR: estimated Glomerular filtration rate; S: Serum; U: urinary; S-Ca: Serum Calcium; U-Ca: urinary calcium; iCa: ionized Calcium; S-Alb: Serum Albumin; UACR: urinary albumin-creatinine ratio; U-Alb: Micro albuminuria.

The mean values are assigned from the standard error to the mean (X ± ESM). The degree of significance is calculated for a risk of error *α* = 5%. The mean comparison is established for each group Whites SS and Blacks SS. Baseline data were obtained before vitamin D supplementation. *p* Value is calculated at baseline time (Whites SS *versus* Blacks SS groups).

#### Estimated glomerular filtration rate (eGFR) status

The black SS subjects show significantly lower eGFR compared to white SS subjects. 25OHD-S appears to significantly enhance the rise in eGFR and slow its decline in both groups. This rise is significant at D2-36 weeks (+21%, *p* < 0.01) in White SS group and increases significantly at D60-36 weeks (+26%, *p* < 0.01) in the Black SS group.

#### Blood and urinary albumin, creatinine, calcium, phosphorus, uric acid, and electrolytes status

At baseline, the data reported in Tables 4 and 5 show that serum creatinine and uric acid were significantly increased in SS Blacks compared to SS Whites. Nonetheless, serum albumin and calcium levels were moderately decreased in black SS compared to white SS. Serum sodium, potassium, and phosphorus levels were comparable between the two SS groups. Black SS subjects had significantly higher levels of urinary albumin, creatinine, uric acid, and ionized calcium compared to white SS subjects. Similarly, the urinary balance sheet also shows a reduction in the excess of sodium, potassium, phosphorus, and calcium in the Black SS group compared to the White SS group. It is important to emphasize that the urinary Sodium-to-Potassium ratio was significantly higher in the Black SS group compared to White SS group. On the other hand, the urinary albumin to creatinine ratio (UACR) was significantly lower in black SS than in white SS. At opposite, the urine creatinine-to-BMI ratio was significantly elevated in black SS *versus* white SS. The 25OHD-S exerted a significant reduction in serum creatinine, creatinine-BMI ratio, and uric acid in both groups. The inhibitory 25OHD-S effect is significant at D2-36 weeks in the whites SS group (−22, −16, and −28%, respectively, *p* < 0.01). Likewise, 25OHD-S decreased significantly the same parameters at D60-36 weeks in the Black SS group (−34, −22, and −29%, respectively, *p* < 0.01). Contrariwise, the 25OHD-S increases significantly serum albumin, calcium, and ionized calcium at D2-36 weeks in whites SS group (+20, +7, and +15%, respectively, *p* < 0.02) and improved the same parameters at D60-36 weeks in Black SS group (+26, +35, and +14%, respectively, *p* < 0.01). Concerning serum sodium, potassium, and phosphorus levels; the difference is not significant before and after 25OHD-S in both groups. The 24-h urine creatinine, sodium, potassium, calcium, uric acid levels and urine volume augmented significantly after 25OHD-S in both SS groups (*p* < 0.001). The excretion of these urinary metabolites is more increase at D2-36 weeks in the whites SS group; while it is more significant at D60-36 weeks in the black SS (*p* < 0.001). At opposite, 25OHD-S declined significantly urinary albumin, UAC ratio in both SS groups (*p* < 0.001). This drop is significant at D2-36 weeks in whites SS group (−26 and −35%, respectively, *p* < 0.001); and significantly decreased at D60-36 weeks in Black SS group (−42 and −48%, respectively, *p* < 0.001). Relating to urinary phosphorus, the difference is not significant before and after 25OHD-S in both groups.

#### Plasma NT-proBNP and cTnT status

**Figure 2.**
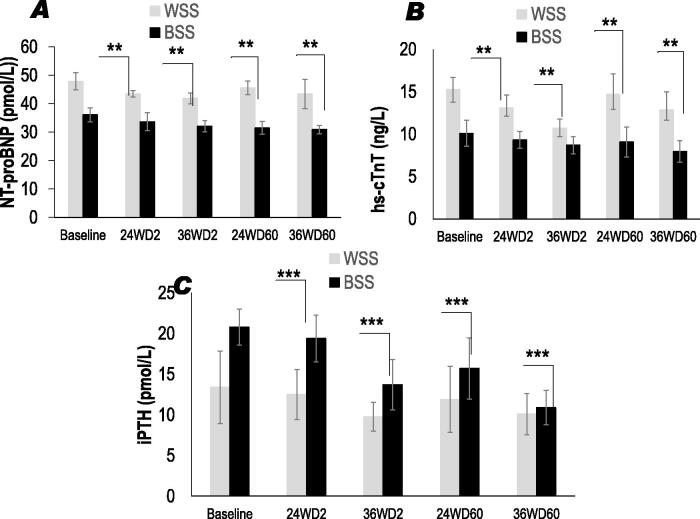
Effects of oral 2000 IU/day and 60,000 IU/month vitamin D3 supplementation on plasma NT-proBNP: N-terminal (NT)-pro hormone (Figure 2(A)); plasma hs-cTnT: high-sensitivity cardiac troponin T (Figure 2(B)); iPTH: intact parathyroid hormone (Figure 2(C)) in CKD Stage 3 Whites and Blacks SS people at 2000 IU doses every day (D2 group) *versus* 60,000 IU doses every month (D60 group) during 24 and 36 weeks vitamin D3 treatment. **p < 0.01; ***p < 0.001.

The baseline data presented in Figure 2(A) shows that plasma N*-*terminal pro hormone (NT-proBNP) levels were significantly reduced in Black SS group compared to whites SS group. Similarly, plasma cardiac cTnT levels were significantly lower in Black SS group compared to whites SS group (Figure 2(B)). Plasma NT-proBNP levels were positively correlated to SBP (*r* = +0.57, *p* < 0.01) and to plasma cardiac cTnT (*r* = +0.39, *p* < 0.01) among black SS participants, but not in whites SS group. Inversely, NT-proBNP was not correlated with eGFR or albuminuria in both groups. Besides, we not found a correlation between plasma NT-proBNP and plasma homocysteine or lipoprotein (a) in both groups. Likewise, no correlation was found between plasma cTnT and plasma homocysteine or lipoprotein (a) in both groups. Interestingly, a significant reduction in plasma NT-proBNP levels was observed after 25OHD-S in both groups (Figure 2(A)). The same effect of 25OHD-S was noted with cTnT in both groups (Figure 2(B)). Nonetheless, this diminution is significant at D2-36 weeks in the whites SS group (−12%) and significantly reduced at D60-36 weeks in the Black SS group (−14%, *p* < 0.02).

### Effects of oral 25OHD-S on iPTH and RAAS profile

#### Plasma total intact parathyroid hormone (iPTH) status

Figure 2(C) indicates that blacks SS showed significantly plasma higher iPTH concentrations compared to whites SS. These results highlight that both groups are affected by SHPT defined as an iPTH > 65 pg/mL, but more accentuated in blacks SS participant than white SS participant. We found a significant inverse correlation between the decrease of eGFR and an increased of iPTH in both groups (*r* = −0.92, *p* < 0.001). In addition, plasma elevated iPTH levels were strongly and inversely correlated with total 25(OH)D deficiency in both groups, but more marked in the black SS than White SS group (*r* = −0.87, *p* < 0.001; *r* = −0.69, *p* < 0.01, respectively). Plasma iPTH accretion declined after 24 and 36 weeks 25OHD-S in both groups. Nevertheless, iPTH levels are significantly decreased (−34%, *p* < 0.001) at D2-36 weeks in the White SS group but did not reach significance in the black SS participants, and SHP persisted in this group. Plasma iPTH concentrations become significantly reduced until dose D60 and at 36 weeks by 47% (*p* < 0.001) in the Black SS group (Figure 2(C)).

#### Plasma levels of renin, aldosterone, and angiotensin II

**Figure 3.**
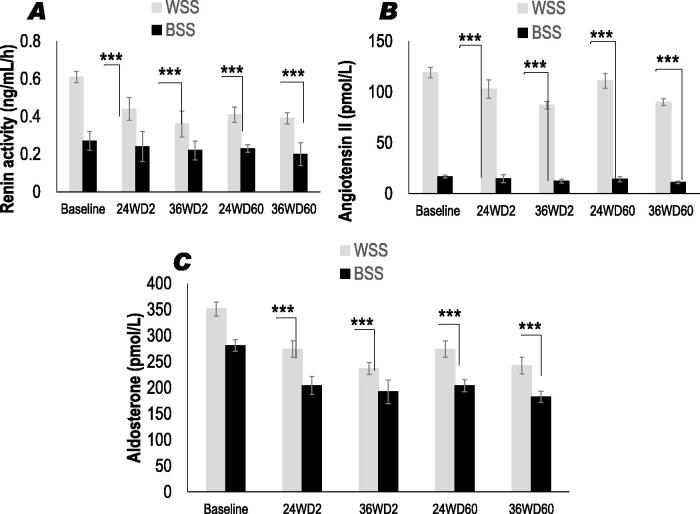
Effects of oral 2000 IU/day and 60,000 IU/month vitamin D3 supplementation on plasma Renin activity (Figure 3(A)); plasma Angiotensin II (Figure 3(B)); plasma Aldosterone (Figure 3(C)) in CKD Stage 3 Whites and Blacks SS people at 2000 IU doses every day (D2 group) *versus* 60,000 IU doses every month (D60 group) during 24 and 36 weeks vitamin D3 treatment. ***p < 0.001.

Figure 3(A) shows that plasma renin activity is significantly lower in black SS compared to whites SS. Similarly, plasma angiotensin II (Figure 3(B)) and aldosterone (Figure 3(C)) were significantly lowers in black SS compared to whites SS. It is important to note that black SS participants were not treated with angiotensin-converting enzyme inhibitors (ACEIs). Our data shown the significant inverse correlation between the urinary sodium and the decrease plasma aldosterone, but not with plasma renin activity (*r* = − 0.44, *p* < 0.01). This correlation has been observed only in Black SS group, but not in Whites SS group. The 25OHD-S leads to a decrease plasma renin activity (Figure 3(A)), angiotensin II (Figure 3(B)), and aldosterone (Figure 3(C)) levels in both groups, but less marked in blacks SS compared to whites SS. At D2-36 weeks, a significant reduction was seen only in the whites SS group (−40, −32, and −27%, respectively, *p* < 0.001). Conversely, a significant decrease was been observed at D60-36 weeks in Black SS groups (−47, −35, and −34%, respectively, *p* < 0.001).

### Effects of oral 25OHD-S on atherogenicity risk

The baseline data on the atherothrombogenic profile are summarized in Tables 2 and 3. The hyperhomocysteinemia is moderate in White SS group, contrary it is slightly higher in Black SS group. Lipoprotein (a) serum levels are abnormally elevated only in the Black SS group compared to the White SS group. This study revealed a significant inverse correlation between total serum homocysteine levels and plasma angiotensin II in the Black SS group (*r* = −0.37, *p* < 0.02). Conversely, hyperhomocysteinemia is not correlated with systolic or diastolic BP in both groups. A significant positive correlation is observed between higher Lp(a) levels and sturdily reduced eGFR in Black SS group (*r* = +0.58, *p* < 0.01), but not in White SS group. As regards Apolipoprotein A1 and B100 (Tables 2 and 3), we did not observe any significant difference between Black and white groups. Nevertheless, it appears that ApoB 100 levels were slightly higher in the Black SS group than in the White SS group. It is important to note a significant positive correlation between Lp(a) increase and elevated Apo B_100_ − Apo A_1_ ratio *versus* to White SS group (*r* = +0.39, *p* < 0.01). Unpredictably, 25OHD-S did not attenuate atherogenicity risk. The serum lipoproteinemia (a) and homocysteine levels persist high (>0.30 g/L, >12 µmol/L, respectively). Interestingly, 25OHD-S decreases significantly Apo B_100 −_ Apo A_1_ ratio in Black SS group at D60-36 weeks (−31%, *p* < 0.001); but moderately in Whites SS group at D2-36 weeks (−17%, *p* < 0.001). The 25OHD-S effect is exerted by reduction of Apolipoprotein B100 (−21 and −9%, respectively, in black and whites SS groups) and elevation of Apolipoprotein A1 (+13%, +10%, respectively, in black and whites SS groups).

## Discussion

In this clinical study, we proved that cholecalciferol supplementation (25OHD-S) influences the components of CMet syndrome, clusters of CRenal syndrome, and RAAS profile, excluding atherogenicity risk. The 25OHD-S effects depend to the racial-ethnic factor. Indeed, it appears that 25OHD-S provides a significant benefit in black and white SS participants by stabilizing the CKD Stage 3 and to prevent its progression to end-stage renal disease. Nevertheless, this study highlight that intermittent high dose (60,000 IU/month) during 36 weeks (long time) in black SS is efficient compared to white SS subjects with lower effective dose (2000 IU/day) during 24 weeks (short time). The difference between black and White SS groups according the supplemented dose and the treatment duration can be explained by several factors which can modulate by the effects physiological of vitamin D: (i) bioavailability of 25OHD; (ii) half-life of serum 25OHD and its steady state; (iii) 25OHD transport *via* chylomicron digestion and its metabolism; (iv) metabolic and vascular factors of target organs; (v) 25OHD signaling pathways *via* the vitamin D receptor [37]. It is described that the prevalence of hypovitaminosis D was common in southern North Africa. Doses higher than 2000 IU/d were necessary to correct hypovitaminosis. On the other hand, in the North Africa regions, a dose of 600 IU/d is sufficient to reach a physiological level with 25OHD ≥30 ng/mL [38]. Among the some arguments, many authors postulate that 25OHD deficiency in black people is associated with increased melanin and declined dermal synthesis of vitamin D from 7-dehydrocholesterol. This seems to be due to enhanced light energy photons absorption by skin. However, this affirmation remains hypothetical and the clinical implications are not confirmed [39]. However, in dark skin subjects (black people); melanin exerts a natural UV-B radiation absorber, and requires greater exposure to UV-B rays than light-skinned subject (white people) to synthesize an adequate amount of 25OH D (vitamin D3) [40]. These studies reveal that dark-skinned SS people are more likely to develop 25OH D deficiency in the absence of adequate sun exposure [41]. Moreover, this difference could result from a feedback inhibition of hepatic 25-OHD synthesis by the elevation of circulating 1,25(OH)2D in black SS residents. [42]. Regarding vitamin D supplementation, some authors have tested several doses as therapeutic targets to limit the CKD progression to dialysis stage [43]. It is important to note that currently no international consensus has been established on the optimal dose and vitamin D duration treatment in CKD [44]. Several doses and duration (higher *versus* lower; long *versus* short) of 25OHD-S to be used in the American black *versus* American white population to study the vitamin D metabolic effects [45]. Besides skin colors and ethnicities, there are some factors that have been demonstrated to have a direct effect on vitamin D status, i.e. sun exposure, latitude, lifestyles, and urbanization. Moreover, serum levels of vitamin D can change over the different seasons in the CKD population. Indeed, relevant studies have shown that levels of 25(OH)D varied by season peaking in autumn with a nadir in spring and calcitriol levels followed a similar seasonal trend. Bone mineral density Z-scores differed between summer and winter at the lumbar spine with a similar trend at the hip. Osteocalcin levels also showed seasonal periodicity and together with alkaline phosphatase were higher in summer than winter [46]. Using plasma PTH levels and urine calcium as metabolic parameters of CKD evolution, some authors have tested high doses of vitamin D (500,000 IU/months) in CKD deficient subjects; while several studies reporting that a single oral high dose of 300,000 − 600,000 IU of D3/months rapidly enhances serum 25OHD [47]. Regarding duration treatment, some authors have shown a rapid increase serum 25OHD after 7 d for high doses. The 25OHD half-life is maintained at 30 d and then drops rapidly. The investigators consider that 25OHD-S can lead to a short half-life (10 − 27 d) with a peak between the 13th and 21st day until the 30th day [48]. Previous studies have experimented a single oral medium dose (100,000 IU/months), the authors observe an increase serum 25OHD levels after 30 d, but decreased slowly until 84 d below 75 nmol/L in serum 25OHD [49]. Alternatively, at lower doses (10,000 − 50,000 IU/months), the vitamin D effects are observed in the long term (3 − 5 months) to reach a 25OHD steady-state [50]. Regarding the 25OHD-S duration, it is essential to emphasize that the vitamin D effects do not observe before 3 months; because a steady state of 25OHD is reached at 3 months [51]. It is for this reason in our study we continued the treatment between 6 and 9 months (24 − 36 weeks). Our study revealed in black SS subjects a drastic depletion of serum 25OHD levels compared to white SS subjects. Our data are corroborated with the epidemiological surveys conducted on the American black population such as the African American Study of Kidney Disease and Hypertension [52]. In our study, the serum 25OHD level normalization is observed concomitantly with normalization of SHPT. It is important to emphasize that black subject requires a high nutritional vitamin D intake close to therapeutic doses, greater than 800 IU/d to correct vitamin D deficiency and obtain physiological effects. At the opposite, white subject requires lower doses [53]. Probably, blacks SS participants requiring an elevated benefit 25OHD-S dose in long period compared to whites SS due to their high plasma PTH levels [54]. In addition, previous studies have showed that parathyroid glands of autopsy black people weighed more than those of white individuals people. These findings provide evidence that SHPT occurs in black’s population [55]. In contrast, bone sensitivity to the PTH action is decreased, which would explain the higher bone mass and the lower osteoporotic fractures prevalence observed in blacks subjects [56]. Interestingly, some authors have shown that an increase PTH level among black’s subjects was independent of serum calcium, phosphorus, 25OHD, and 1,25OHD levels. These authors suggest that other mechanisms may play a role in the development of SHPT in black’s people. It is possible that this is related to the expression or activity of calcium-sensing receptors in the parathyroid glands. Indeed, the activation of calcium-sensing receptors by increased ionized calcium inhibits PTH secretion, and decreased expression of calcium-sensing receptors is a mechanism of SHPT in black CKD. The HOMA-IR is found more acute in the Black SS group compared to White SS group, which partly explains the high BP common in the black population. It is admitted that insulin is involved in vasoconstrictive signaling pathways of endothelin, including the pathways of (extracellular signal-regulated kinase (ERK) and mitogen-activated phosphate kinase (MAPK) [57]. Regarding anthropometric status, we observed that 25OHD-S promotes weight diminution related to abdominal fat loss and to attenuate obesity in Black SS groups [58]. In our study, the decrease in bioavailability of circulating 25OHD leading to vitamin D deficiency in obese black SS participants may be due to vitamin D sequestration in adipose tissue [59]. In our study, the 25OHD-S effects have improved insulin resistance and insulin sensitivity in peripheral tissues, attested by the HOMA index attenuation [60]. Likewise, the 25OHD-S effect has reduced glucose intolerance and dyslipidemia. It is described that vitamin D exerts its effects *via* the vitamin D responsive element (VDRE) signaling in the *β* pancreas cells, which improves the insulin secretion [61] and insulin resistance [62,63] *via* metabolic calcium signaling pathways [64]. Regarding lipid metabolism, 25OHD-S inhibits lipogenesis *via* decreased the fatty acid synthetase activity, and increased lipolysis *via* the phospholipase C and cAMP activation [65]. However, some authors suggest that 25OHD-S may indirectly lead to lipolytic effects by inhibiting SHPT. This hormonal disorder is described as the major cause of weight gain in 25OHD deficient CKD patients [66]. In our study, after 25OHD-S, we have shown that SHPT is attenuated. This allows an intracellular modulation by calcium in adipocytes *via* the VDRE signaling which inhibits PTH gene transcription [67]. Besides, it is important to note that Vitamin D activates directly the transcription factor peroxisome proliferator-activated receptor-γ (PPAR-γ) which is implicated in the regulation of fatty acid metabolism in muscle and adipose tissue [68]. On the other hand, we have shown that serum HDL-C are higher and TG levels are significantly reduced in blacks SS *versus* whites SS participants. We speculate that they may be related to the variations in activity of plasma enzymes allowing TG storage from plasma to peripheral tissues, and therefore to modulate the lipoproteins composition, such as lipoprotein lipase (LPL). LPL activity has been shown to be higher in black’s people compared to White’s people [69]. Interestingly, black’s individuals were not accompanied by drastically perturbed lipid profile, as is frequently found in White’s populations. Many studies have been described the association of vitamin D with hypertension and cardiovascular risk [70], predominantly with its crucial regulator effects throughout elevated Renin-angiotensin-aldosterone system (RAAS) activity. Indeed, experimental studies have shown that during elevation in circulating RAAS components, VDR-null mice display increased expression of renal-vascular renin mRNA [71], supporting experiments that suggest that vitamin D may be an inhibitor of renin gene expression [72]. In our study, we observed that 25OHD-S modulates BP by inhibiting the increase systolic BP, but not diastolic BP. Some authors explain this effect by low renin levels [73] to lead vascular effects, since the vascular endothelium expresses the VDRE [74]. On the other hand, the hypertrophy of abdominal adiposity observed in black SS participants, contributes to the BP disorder, since it is admitted that adipose tissue is an important source of production of Angiotensin II, a powerful vasoconstriction factor [75]. Besides, it is described that SHPT contributes to increase BP, myocardial hypertrophy, and ventricular arrhythmias [76]. In our study, the 25OHD-S may contribute to lowering hypertension after SHPT attenuation in black SS participants. On the other hand, some authors hypothesized that vitamin D was involved in regulating the flux of calcium into vascular smooth-muscle cells, thereby influencing intracellular calcium concentrations, vascular tone, and BP [77] and decreasing renin secretion from juxtaglomerular cells [78]. Previous studies, according vitamin D deficiency in experimental renal injury by obstructed kidney [79], authors have shown an attenuation of renal fibrosis after an angiotensin receptor antagonist treatment. These results suggest signaling defect through the VDR and resulted in unfavorably high intrarenal RAAS activity. Similarly, animal model deficiency in the 1-alpha-hydroxylase activity exhibited increased activity of the intra-renal RAAS. This deficiency can be ameliorated with 1,25(OH) 2 D treatment (paricalcitol, a VDR agonist) [80]. The most important point in this study is undoubtedly the attenuation of the eGFR decline. In this study, the 25OHD-S has a significant inhibition of decrease the eGFR in both SS groups. In agreement with our findings, some authors have highlighted 25OHD exerts its effects by reducing hypercreatininemia in nephron tubular [81]. It is important to emphasize that vitamin D deficiency is involved in glomerulonephritis *via* its role on immunomodulation, inflammatory, and autoimmune processes. Vitamin D supplementation seems to significantly reduce proteinuria and to slow kidney disease progression in glomerulonephritis. It also has potent antiproliferative and immunomodulating functions, contributing to the inhibitions of kidney inflammation. Vitamin D preserves the structural integrity of the slit diaphragm guaranteeing protective effects on podocytes [82]. In our study, the 25OHD-S promotes the creatinine excretion. This question is poorly documented; however, the hypothesis would be that 25OHD-S increases creatinine clearance by extra renal pathway, such as intestinal excretion (digestive creatinine source). The 25OHD-S can stimulate the microbiota [83,84] inducing the creatininase activity; particularly by Escherichia coli bacterium that expresses the creatininase gene [85]. In black and whites SS, the intestinal creatinine degradation mechanism by which 25OHD activates microbiota remains unclear. The presence of microalbuminuria in the Blacks SS group is related to dyslipidemia by the non-esterified free fatty acids (NEFFA) albumin transport. The impaired kidney function leads to a decrease the glomerular albumin ultrafiltration. Since albumin is continuously saturated with excess circulating NEFFA, which leads to proteinuria [86]. A recent study showed that vitamin D supplementation inhibits the progression, particularly microalbuminuria of diabetic nephropathy and increased cardiovascular risk factors *via* the modulation of RAAS componements [87]. This effect is explained by the megalin pathway, a protein (multi-ligand receptor) which promotes tubular albumin reabsorption by receptor-mediated endocytosis of the VDRE-25(OH) D complex, which leads to restore megalin synthesis [88]. In addition, megalin pathway is involved in the vitamin D reuptake in the proximal tube [89]. It is noted that despite a much lower serum 25OHD level in the Black SS group, calcium metabolic profile appears to be more efficient and improved bioavailable than white SS participants. Our data are in agreement with other recent studies which have shown that better calcium absorption was significantly correlated with serum 25OHD but also with intestinal transit and the urinary calcium/creatinine ratio. Moreover, the decrease in urinary calcium in black SS participants results from a reduce in intestinal calcium absorption linked to the increase in serum PTH, serum 1,25(OH)2D, and possibly enhances in urinary cyclic adenosine monophosphate (cAMP). Surprisingly, increased circulating PTH in black’s people seems to prevent calcium urinary loss *via* enhancing the tubular reabsorption [90]. Regarding potassium profile, in our study we have noted a significative reduction of urinary potassium in blacks compared to white’s subjects. It is interesting to underline that our results agree with some studies which observed a low plasma level of renin and aldosterone in black subjects [91]. Probably, this can be explained by a decrease in intestinal absorption of phosphate and potassium in black SS participants compared to white SS. In addition, another argument could explain that the increase in both plasma PTH and 1,25(OH)2D, and the decrease in urinary calcium is the consequence of a inhibition in the calcium intestinal absorption. In this context, our data can be supported by the activation of RAAS mediators which has been proposed to argue this racial disparity in black population observed in the incidence of hypertension and cardio-renal complications, including Ang II and aldosterone [92]. Indeed, the hypersecretion of aldosterone as a potential risk factor for cardiovascular events in the CKD population, in particular, the higher rate of hypertension-related in Algerian SS blacks [93]. The authors seem to attribute this disorder to increase on dietary salt. In fact, previous studies have shown that the Algerian blacks consumed more salt than the Algerian whites in Sahara [94]. Several mechanisms have been suggested to explain the sodium retention and increased salt sensitivity in black population, such as the gene mutation encoding the epithelial sodium channel (ENaC) of the collecting tubule, thereby causing increased sodium reabsorption. The ENaC channel is under the aldosterone control which stimulates its expression [95]. We suppose that black SS CKD participants have a reduced ability to excrete high sodium intake, which interferes in the sodium balance increasing the CRenal biomarkers. Indeed, the angiotensin II and aldosterone effects are known to be amplified by a high salt intake by increased transforming growth factor (TGF-β) expression in kidney and aortic endothelial cells, impacting the cardiovascular function and renal structure. On the other hand, low plasma renin levels in black SS compared to white SS participants, are argued by different factors, include high renal sodium reabsorption, reduced conversion of pro-renin to renin, and lower soluble plasma renin receptor [96]. The suppression of renin secretion in black SS is explained by volume loading who associated with increased sodium retention, which may result the water kidneys retention. When water is retained it may lead to expansion of the extracellular fluid volume and thereby increasing BP. In turn, this may lead to even further plasma renin depletion [97]. Our investigation highlights 25OHD deficiency and increased plasma levels of cTnT and NT-proBNP. Several previous studies have found that elevated plasma NT-proBNP levels predict heart failure in ESRD patients [98] but not in stage 3 CKD (our study). In our opinion, 25OHD deficiency affects vascular smooth muscle, as endothelial cells express VDR and can subsequently lead to an increase in cTnT [99]. Some experimental studies have shown that calcitriol supplementation may suppress myocardial hypertrophy by inhibition endothelin synthesis [100]. Besides, Vitamin D stimulates nitric oxide (NO) production in a CKD and therefore allows endothelial relaxation. Such mechanism is related to a dose-dependent activation of VDR that triggers endothelial nitric oxide synthase (eNOS) phosphorylation *via* p38 and protein kinase B (Akt) [101]. Likewise, experimental studies have shown that Vitamin D promote vascular regeneration *via* to increase the number of angiogenic myeloid cells and to stimulate the re-endothelialization [102]. Regarding the homocysteine, it was described very recently an interaction between vitamin D deficiency, increased serum homocysteine levels and coronary artery disease *via* VDR dysfunction. Additionally, previous longitudinal study showed a relationship between the hyperhomocysteinemia and the development of CKD [103]. This is mainly attributed to decreased homocysteine renal filtration since nephrons function decline. However, elevated homocysteine might be an independent predictor risk factor for the development of heart failure during CKD progression [104]. It is described that hyperhomocysteinemia may decrease adenosine in interstitial tissue, and induce glomerular mesangial apoptosis, which leads to renal vascular injury *via* activation of p38-mitogen-activated protein kinase [105]. In our study, the 25OHD-S enhances the thrombogenic effect of hyperhomocysteinemia, *via* a vascular prevention disease and anti-atherosclerotic venous thrombosis. Indeed, some studies have shown that supplementation with 25(OH)D allows to reduce hyperhomocysteinemia among CKD patients, but in subjects who have serum 25(OH)D levels less at 21 ng/mL [106]. Nevertheless, we cannot confirm whether 25OHD-S reduces homocysteine levels by itself *via* VDR or by reducing iPTH. In this study, we found that Lp(a) levels are abnormal. This observation can be argued by the eGFR drop which increases the Lp(a) levels, which is eliminated essentially by glomerular filtration [107]. Otherwise, some studies have highlighted the association between increased Lp(a) levels and essential hypertension *via* renal endothelial dysfunction. Previous work has shown that Lp (a) can be sequestered in the arterial subintimal space much more than oxidized LDL-c, and therefore lead to greater atherogenicity [108]. It has been proposed that Lp (a) promotes the vascular dysfunction by decreasing the concentrations of NO [109]. In addition, it is important to emphasize that Black SS group showed an acute ApoB100/ApoA1 ratio *versus* White SS group. In this context, numerous experimental studies have shown a relationship between the activation of VDR and increased expression of ApoA1, through modulation of sterol responsive element binding protein 2 (SREBP2) [110]. Although vitamin D and Lp (a) were independently associated, the 25OHD-S attenuate atherothrombogenic risk by normalizing hyperlipoproteinemia (a) in Whites SS group but not in Black SS group. Some authors suggest that 25OHD-S decrease serum lipoprotein (a) *via* increase apoprotein A1 [111] which explain the effects of 25OHD-S on decreasing the Apo B_100 −_ Apo A_1_ ratio in the Black SS group.

## Conclusion

**Figure 4.**
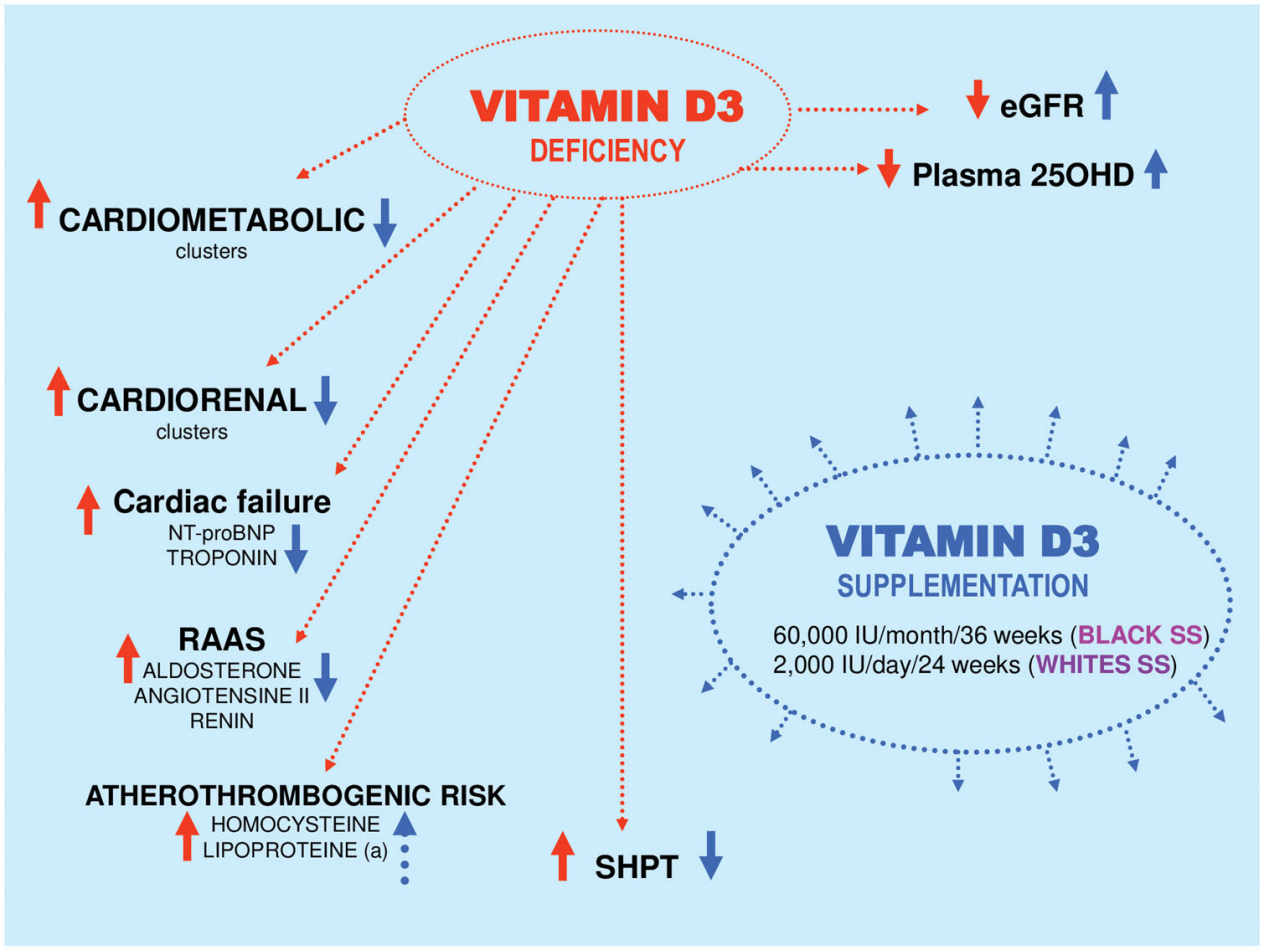
Summary of modulation of cardiometabolic risk and CardioRenal syndrome by oral vitamin D_3_ supplementation in Black and White Southern Sahara Residents with chronic kidney disease Stage 3.

This study highlights the importance of the racial-ethnic factor during vitamin D_3_ (Cholecalciferol) supplementation in CKD subject. It appears that 25OHD-S provides a significant benefit in black compared to white SS participants by stabilizing the renal disease stage 3 by inhibiting the deleterious effects both CRenal and CMet syndrome clusters to prevent its progression to end-stage renal failure and renal dialysis (Figure 4). In perspective, it would be interesting to explore the genetic metabolome of Black people *versus* White subjects, particularly renal function.

## Limitations of this study

Limitations of this study are the small number of participants, the short duration of intervention and we enrolled only CKD Stage 3. It was also necessary to take CKD Stage 4 to confirm the effectiveness of vitamin D.

## Informed consent statement and consent for publication

Informed consent was obtained from all participants involved in the study. All authors listed have approved the manuscript for publication.

## Data Availability

All data presented in this study are available on request from the corresponding author on reasonable request.

## Abbreviations

25OHD-S: cholecalciferol supplementation
BMI: body mass index
cAMP: cyclic adenosine monophosphate
CKD: chronic kidney disease
CKD-EPI: chronic kidney disease – EPIdemiology collaboration
CMet: cardiometabolic
CRenal: cardiorenal
cTnT: Troponin T
CVD: cardiovascular disease
D2: Dose of 2,000 IU of cholecalciferol /day/24 weeks
D60: Dose of 60,000 IU of cholecalciferol /day/36 weeks
DBP: diastolic blood pressure
eGFR: estimate glomerular filtration rate
ENaC: epithelial sodium channel
eNOS: endothelial nitric oxide synthase
ESRD: end-stage renal disease
FGF-23: Fibroblast Growth Factor-23
HOMA-IR: homeostasis model assessment insulin resistance
hs-cTnT: high-sensitivity cardiac troponin T
iPTH: intact parathyroid hormone
KDIGO: Kidney Disease Improving Global Outcome
Lp (a): lipoprotein(a)
NCEP/ATPIII: National Cholesterol Education Program Third Adult Treatment Panel
NEFFA: non-esterified free fatty acids
NF-κB: nuclear factor-kappaB
NKF-KDOQI: National Kidney Foundation - Kidney Disease Outcomes Quality Initiative
NO: nitric oxide
NT-proBNP: N-terminal pro B-type natriuretic peptide
PPAR-g: peroxisome proliferator-activated receptor-g
RAAS: Renin-angiotensin-aldosterone system
SBP: systolic blood pressure
SHPT: secondary hyperparathyroidism
SREBP2: sterol responsive element binding protein 2
SS: Southern Sahara
tHcy: total homocysteine
VDR: vitamin D receptor
VDRE: vitamin D response element
UACR: urinary albumin-to-creatinine ratio
UCr: Fasting urinary Creatinine
WC: waist circumference
